# Complement Fixation

**Published:** 1920-10-09

**Authors:** 


### Complement Fixation.

### THE CLINICAL TESTS FOR TUBERCULOSIS.

THE search after a sure objective test of active tuberculosis resembles the old one after the philosopher's stone. And it is not hard to see why this should be so. We see (as, for instance, in an illustrated article in Brauer's *Beiträge zur Klinik der Tuberkulose*, *April* 1920) that in a savage community, into which the disease has been recently introduced, the matter is quite simple. You apply v. Pirquet's test to the arms of all suspects; and in all infected cases a vigorous reaction early makes its appearance. These people, as clinical experience shows repeatedly, are serious cases; soon they will be gravely ill—the greater part of them, indeed, will die. They want removing from the rest of the community. But the non-reactors are certainly free from tuberculosis. In a word, your savage either has serious disease or he has none. There is no betwixt and between, no latency, no minimal lesions which give the test but need no treatment.

METHODS DISCARDED.

Now in western civilised communities the sources of error and failure just enumerated do obtain abundantly. On this account the v. Pirquet and allied tests have been largely given up, except in the case of nursing children, who for purposes of tuberculosis, as well as in other things, are like savages. And hitherto the complement fixation test has failed on account of error, not in excess, but in defect. For some reason the blood in tuberculosis is much less sensitive to it than in syphilis, and thus many negative reactors to the tubercle complement fixation test (even with its buttresses of previous antigen treatment) may turn out to be tuberculosis after all.

THE OBVIOUS RESPONSE.

The obvious response on the part of investigators to this state of things is to modify the technique of the test in the direction of magnification of sensibility. It does not need much acumen to see that, then, in steering clear of the rocks they may be approaching the whirlpool; and the latter state of the complement fixation test come to approach the present one of the v. Pirquet reaction. In truth, the infinite variety of tuberculous infection, from minimal to massive, in civilised man, will always make it very difficult to devise a test objective and scientific, which shall settle a vastly important but still purely arbitrary point, as to whether the patient is infected in the clinical sense of the word, or no.

